# Urinary Exosomal MicroRNAs in Incipient Diabetic Nephropathy

**DOI:** 10.1371/journal.pone.0073798

**Published:** 2013-11-04

**Authors:** Federica Barutta, Marinella Tricarico, Alessandro Corbelli, Laura Annaratone, Silvia Pinach, Serena Grimaldi, Graziella Bruno, Daniela Cimino, Daniela Taverna, Maria Chiara Deregibus, Maria Pia Rastaldi, Paolo Cavallo Perin, Gabriella Gruden

**Affiliations:** 1 Diabetic Nephropathy Laboratory, Department of Medical Science, University of Turin, Turin, Italy; 2 Renal Research Laboratory, Fondazione IRCCS, Ospedale Maggiore Policlinico and Fondazione D’Amico per la Ricerca sulle Malattie Renali, Milan, Italy; 3 MIA Consortium for Image Analysis, Milano Bicocca University, Milan, Italy; 4 Department of Biomedical Science and Human Oncology, University of Turin, Turin, Italy; 5 Molecular Biotechnology Center (MBC), University of Turin, Turin, Italy; 6 Laboratory of Renal and Vascular Pathophysiology, Department of Medical Science, University of Turin, Turin, Italy; IRCCS-Policlinico San Donato, Italy

## Abstract

MicroRNAs (miRNAs), a class of small non-protein-encoding RNAs, regulate gene expression via suppression of target mRNAs. MiRNAs are present in body fluids in a remarkable stable form as packaged in microvesicles of endocytic origin, named exosomes. In the present study, we have assessed miRNA expression in urinary exosomes from type 1 diabetic patients with and without incipient diabetic nephropathy. Results showed that miR-130a and miR-145 were enriched, while miR-155 and miR-424 reduced in urinary exosomes from patients with microalbuminuria. Similarly, in an animal model of early experimental diabetic nephropathy, urinary exosomal miR-145 levels were increased and this was paralleled by miR-145 overexpression within the glomeruli. Exposure of cultured mesangial cells to high glucose increased miR-145 content in both mesangial cells and mesangial cells-derived exosomes, providing a potential mechanism for diabetes-induced miR-145 overexpression. In conclusion, urinary exosomal miRNA content is altered in type 1 diabetic patients with incipient diabetic nephropathy and miR-145 may represent a novel candidate biomarker/player in the complication.

## Introduction

Diabetic nephropathy (DN) is a major cause of renal replacement therapy in the Western World. Microalbuminuria is widely used as a biomarker for DN; however, its clinical relevance as a surrogate outcome in chronic kidney disease has not been confirmed and recent studies suggest that microalbuminuria is a less precise predictor of nephropathy risk than originally thought [1-3]. There is, thus, an increasing quest to find novel biomarkers to identify and treat individuals at high risk. In addition, early biomarker discovery may also help to identify new players in the pathogenesis of the glomerular injury in diabetes.

Urine is an ideal source of biomarkers for renal diseases [4] and urinary mRNA profiling has been proposed as a tool for biomarker discovery. However, urinary mRNAs are labile as easily degraded by urinary RNase and they often originate from apoptotic/necrotic cells with a scarcely representative transcriptional profile [4]. Urine also contains cup-shaped vesicles, known as exosomes, which derive from the cellular endocytic compartment [5]. Exosomes carry RNAs that can deliver to distant target cells and represent an important way of cell-to-cell communication [6]. At variance with free urinary RNAs, exosomal nucleic acids are in a remarkable stable form as microvesicles protect them from endogenous RNase activity. Furthermore, they derive from viable cells from all nephron segments and may thus provide valuable insight on renal pathophysiology [4].

Exosomes contain microRNA (miRNA), a class of small non-protein-encoding RNAs that regulate gene expression via suppression of target mRNAs [7]. Specifically, miRNAs bind through canonical base pairing to a complementary site in the 3’ untranslated region of their target mRNAs and can direct the degradation or translational repression of these transcripts [7]. MiRNAs are critically involved in many biological processes and accumulating evidence also points to an important role of miRNAs in the pathogenesis of both diabetes and diabetes-related complications [8,9]. Moreover, miRNAs, present in body fluids, can display unique expression profiles in pathological conditions and it has been proposed that distinctive miRNA signatures may be exploited as innovative diagnostic/prognostic tools [10-12]. Despite the growing interest in miRNAs, urinary exosomal miRNAs have never been studied in either normal or pathological conditions.

In this study, we have assessed miRNA expression in urinary exosomes from type 1 diabetic patients (DM1) with and without incipient DN. Furthermore, pathophysiological relevance of miR-145, which was enriched in urinary exosomes from microalbuminuric patients, was explored in both streptozotocin-induced diabetic mice and cultured mesangial cells.

## Materials and Methods

### Materials

All materials were purchased from Sigma-Aldrich (St Louis, USA) unless otherwise stated.

### Subjects

The study was approved by the Ethical Committee of Turin University, the procedures were in accordance with the Helsinki Declaration, and informed written consent was obtained from all subjects. Twelve DM1 with persistent microalbuminuria and normal renal function and 12 normoalbuminuric DM1 comparable for age, sex, and diabetes duration were consecutively enrolled in the study. Ten non-diabetic subjects similar for demographic characteristics (age: 56.3 ± 2.8; sex: 10/0 M/F; hypertension Y/N: 5/5) served as control group. Patients with cardiovascular diseases, non-diabetic kidney diseases, or renal tract pathological conditions were excluded. Microalbuminuria was defined as either an albumin excretion rate (AER) value of 20-200 μg/min or an urinary albumin/creatinine ratio (ACR) of 2.5-25 mg/mmol in males and 3.5-35 mg/mmol in females in two out of three overnight urine collections. ACR values were converted in AER values using a conversion formula previously developed in DM1 [13]. Hypertension was defined as systolic blood pressure >140 mmHg and/or diastolic blood pressure >90 mmHg or treatment with antihypertensive drugs. Diabetic retinopathy was assessed by direct funduscopic examination. Urinary albumin was measured by nephelometry, urinary creatinine concentration by the modified Jaffe method, and HbA1_C_ by ion-exchange liquid chromatography. 

### Urinary exosomes isolation

Overnight urine collections (^~^450 mL/subject) were obtained from all recruited subjects. Urines were pre-cleared by both centrifugation (300 *g* 10 min at 4 °C and 17,000 *g* 20 min at 4 °C) and filtration (0.8 μm) to remove cellular debris. Then, exosomes were isolated by two consecutive ultracentrifugation steps (200,000 *g* 75 min at 4 °C; 70.1 Ti rotor, Beckman Instruments, Fullerton, CA) as previously described [14]. Pellets were suspended in phosphate buffer and exosome quality and purity assessed by electron microscopy and immunoblotting. Exosome quantity was determined by measuring the rate of Brownian motion using a NanoSight LM10 system, which is equipped with a fast video capture and particle-tracking software (NanoSight Ltd, Wiltshire, UK). In selected experiments, exosomes were treated with RNase A (1U/ml, Fermentas, Glen Burnie, MD) prior to RNA extraction.

### Immunoblotting

Pellets obtained after ultracentrifugation was dissolved in 100 μl of Laemmli buffer. Total protein concentration was determined using the RC DC Protein Assay Kit (Bio-Rad, Milan, Italy). Proteins (^~^70µg) were separated by SDS-PAGE and transferred to nitrocellulose membranes (Amersham Pharmacia, Freiburg, Germany). Following blocking, membranes were incubated with primary antibodies against Hsp70 (StressGen, Victoria, BC Canada), alix, or calnexin (SantaCruz, Glostrup, Denmark) overnight at 4°C. After washing, secondary HRP-linked (SantaCruz) antibodies were added. Detection was performed using either the ECL chemiluminescence substrate (Amersham) or the super signal PICO/FEMTO (Euroclone, Milan, Italy) and visualised on a Gel-Doc system (Bio-Rad, Milan, Italy). Total protein extracts from mesangial, HeLa, and 293T cells were used as positive control for Hsp70, alix, and calnexin, respectively. GAPDH was used as loading control for calnexin.

### Trasmission Electron Microscopy

Pellets resuspended in PBS were loaded onto formwar carbon coated grids, fixed in 4% paraformaldehyde, and washed. Microvesicles were post-fixed in 2.5% glutaraldehyde, washed, contrasted in 2% uranyl acetate, embedded in a mixture of uranyl acetate (0.8%) and methyl cellulose (0.13%), and examined with a Philips CM 10 electron microscope. Microphotographs were taken using a SIS Megaview II digital camera.

### Total RNA extraction and miRNA expression profiling

Total RNA was extracted using the Trizol reagent (Invitrogen, Milan, Italy) and quantified by spectrophotometry (Nanodrop ND-1000 Wilmington DE). On average 1-2 μg of total RNA were obtained from each subject. RNA quality was assessed by capillary electrophoresis on an Agilent-2100 Bioanalyzer (Agilent Technologies, Santa Clara, CA). MiRNAs were reverse transcribed using the Megaplex Primer Pool A, Human Pool A v2.1 (Applied Biosystems), which contains RT primers for 377 miRNAs, 3 endogenous controls, and a negative control. RT reaction products were pre-amplified using the Megaplex PreAmp Primers (Primers A v2.1) and the TaqMan® PreAmp Master Mix. MiRNA expression profile was performed in 2 pairs of micro/normoalbuminuric DM1 patients tightly matched for HbA1c (1^st^ pair: 8.1 vs. 8.1, 2^nd^ pair: 8.7 vs. 8.7) by Human TaqMan miRNA Array A on an 7900HT Fast Real-Time PCR System. Raw Ct values were calculated using the SDS software and standardized to U6 snRNA. MiRNAs were excluded if both samples within a pair had Ct values ≥ 35/undetermined. Relative expression was calculated using the comparative Ct method (2^-ΔΔCt^). MiRNAs were considered differentially expressed if they exhibited greater than twofold expression differences in both pairs. Data are publicly available at the NCBI Gene Expression Omnibus repository (accession number: GSE48318). Interrogation of the www.microrna.org database was performed to identify differentially expressed miRNA known to be expressed by glomerular cells (in silico analysis).

### Taqman qPCR

Expression of a subset of miRNAs (miR-145, miR-424, miR-155, miR-130a) was quantitated in both DM1 and control subjects,using specific Taqman microRNA Assays. Diluted pre-amplification products were combined with Taqman miRNA Assay and Taqman Universal PCR Master Mix No AmpErase UNG, then a qPCR was performed on an Applied Biosystems 7900HT thermocycler. All samples were run in triplicates and standardized to U6 snRNA using the SDS2.2 software. 

### Animals and diabetes induction

The study was approved by the Ethical Committee of the Turin University, and both housing and care of laboratory animals were in accordance with Italian law (D.L.116/1992). Male C57BL6/J mice from Jackson Laboratories (Bar Harbor, ME) were maintained on a normal diet under standard animal house conditions. Diabetes was induced by intraperitoneal injection of streptozotocin (55 mg/kg body wt/day), delivered in five consecutive daily doses. Mice that were sham injected with sodium citrate buffer were used as controls. Diabetes onset was confirmed by blood glucose levels >250 mg/dL 4 weeks after the first dose of STZ. Animals were killed ten weeks after diabetes onset. Prior to sacrifice, blood samples were taken via saphenous vein puncture on alert 4-hours–fasted animals and urine collected. Glucose levels were measured using a glucometer (Roche, Milan, Italy) and glycated hemoglobin by quantitative immunoturbidimetric latex determination (Sentinel Diagnostic, Milan, Italy). 

### Urine collections and exosomal isolation

Urine was collected over 18 hours, with each mouse individually housed in a metabolic cage and provided with food and water ad libitum. Urinary albumin concentration was measured by a mouse albumin ELISA kit (Bethyl Laboratories, Milan, Italy). Urinary exosomes were isolated from pooled urine from both diabetic and non-diabetic mice (n=30 per group) as described above.

#### Glomerular isolation

Both non-diabetic (n=5) and diabetic (n=5) mice were anesthetized and perfused with 8x10^7^ surface-inactivated Dynabeads (Invitrogen, Milan, Italy), the glomeruli were isolated using a modified Takemoto et al method [15,16]. 

### Glomerular and urinary exosomal miR-145 expression

Total RNA was extracted from both urinary exosomes and isolated glomeruli of diabetic and control mice using the Trizol reagent, then miR-145 expression quantitated by Taqman qRT-PCR as described above. 

### Mesangial Cell Culture

Cultures of immortalised human mesangial cells were established and characterized as previously described [17]. Cells were cultured in Dulbecco’s Modified Eagle’s Medium (Invitrogen, Milan, Italy), containing L-glutamine, 6.8 mM glucose, 20% exosomes-depleted foetal calf serum (FCS, Euroclone Milan, Italy) (17), 100 U/ml penicillin, and 100 µg/ml streptomycin in a humidified 5% CO_2_ incubator at 37°C. 

### Mesangial Cell Transfection of miR-145

Mesangial cells were transfected with either a pre-miR-145 precursor (Life Technology) or a negative control using Lipofectamine 2000 (Invitrogen). After 24 hours, cells were lysed and STAT-1 protein expression by immunoblotting using a specific rabbit anti-STAT-1 antibody (Cell Signalling, Milan, Italy). Tubulin was used as loading control.

### Mesangial Cell Exposure to High Glucose Concentration

After serum deprivation for 24 hours, human mesangial cells were exposed to increasing (6.8 mM, 15 mM, and 25 mM) glucose concentrations for various time period (4, 6, 12, and 24 hours). Media were made iso-osmolar with the addition of mannitol. Total RNA was extracted and miR-145 levels measured as described above. At the 24 hours time point mesangial cell supernatants were also collected and exosomes isolated by differential centrifugations. Briefly, supernatants were centrifuged at 300 g for 10 min then at 20,000 g for 20 min, followed by filtration through a 0.22 µm filter. Exosomes were then pelleted by ultracentrifugation at 120,000g for 70 min and total RNA extracted for miR-145 measurement as described above. An aliquot of the exosomal preparation was used for exosome counting by NanoSight LM10 system followed by normalization to total cell number.

### Data presentation and statistical analysis

Data were expressed either as mean ± SEM or as geometric mean (25^th^-75^th^ percentile). Results were analyzed by t test or ANOVA, as appropriate. Variables with skewed distribution were analyzed after logarithmic transformation. Values for P<0.05 were considered statistically significant.

## Results

### Patients characteristics

Clinical and laboratory characteristics of recruited subjects are shown in Table 1. Micro- and normoalbuminuric patients were comparable for age, sex, diabetes duration, and renal function. HbA1_c_ values were greater in micro- than in normoalbuminuric patients, but this did not reach statistical significance. As expected, AER was higher in patients with incipient DN than in normoalbuminuric subjects.

**Table 1:** Clinical and Laboratory Parameters of Type 1 Diabetic Patients.

	**Normoalbuminuric DM1**	**Microalbuminuric DM1**
**N**	12	12
**Age (years)**	57.9 ± 3.7	58.0 ± 2.7
**Sex (male/female)**	12/0	12/0
**Diabetes duration (years)**	33.8 ± 3.5	30.7 ± 2.8
**HbA1_C_ (%)**	7.8 ± 0.3	8.2 ± 0.3
**Serum creatinine (mg/dl)**	0.8 ± 0.02	0.9 ± 0.04
**Retinopathy (y/n)**	8/4	12/0
**Hypertension (y/n)**	7/5	7/5
**AER (μg/min)**	8.00 (6.62-7.67)	72.30 (62.18-89.90)*

Data are means ± SEM; DM1: type 1 diabetic patients, AER: albumin excretion rate; *p<0.001 microalbuminuric vs normoalbuminuric patients.

### Characterization of urinary exosomes

Vesicles, isolated from overnight urine collections, were <100 nm in size and showed a characteristic cup-shaped morphology at the electron microscopy (Figure S1A). Immunoblotting analysis demonstrated expression of both Hsp70 and alix, which are well-established exosomal markers [18]. On the contrary, vesicles were negative for calnexin, an endoplasmatic reticulum protein, confirming the absence of contamination from other cellular compartments (Figure S1B). 

Exosome quantity in overnight urine collections was similar in diabetic patients [335 x 10^8^ (303-562), geometric mean (25^th^-75^th^ percentile)] as compared to controls [251 x 10^8^ (128-700)], a reduction in urinary exosome number was observed in microalbuminuric DM1 [175 x 10^8^ (112-245)], though this did not reach statistical significance.

### Urinary exosomes contains small RNA

Total RNA, extracted from isolated exosomes, was analyzed on an Agilent 2100 Bioanalyzer and the resulting electropherogram showed that urinary exosomes were enriched in small RNA species, including miRNAs, but did not contain ribosomal RNA (Figure S1C). RNA was confined within exosomes as it was still found when intact microvesicles were pre-treated with RNase (Figure S1D). 

### MiR-130a, miR-424, miR-155, miR-145 are differentially expressed in urinary exosomes from microalbuminuric DM1

Two hundred and twenty six miRNAs were detectable in the urinary exosomes from normoalbuminuric DM1 patients. Differential miRNA profiling showed that expression of 22 miRNAs differed in both matched pairs of micro/normoalbuminuric patients. Out of them 4 miRNAs were chosen for further analysis by qRT-PCR in all recruited subjects. MiR-130a, miR-424, miR-155 were selected as they are expressed by cultured human podocytes (www.microrna.org); miR-145 was chosen because is a known marker of glomerular mesangial cells (19,20). Consistent with profiling results, qRT-PCR analysis showed that miR-155 and miR-424 levels were significantly lower, while miR-130a and miR-145 levels significantly higher in micro- than in normoalbuminuric patients. MiR-155 and miR-424 were similar in normoalbuminuric diabetic patients and non-diabetic controls, while levels of miR-145 and miR-130 were greater in normoalbuminuric diabetic patients than in controls, though this did not reach statistical significance (Table 2). 

**Table 2:** MiR-130a, miR-424, miR-155, miR-145 content in urinary exosomes from diabetic patients and non-diabetic control subjects.

	**Non-diabetic Controls**	**Normoalbuminuric DM1**	**Microalbuminuric DM1**
**miR-145**	0.66 (0.26-1.73)	1.14 (0.73-1.71)	3.11 (2.09-5.10)*#
**miR-130a**	0.44 (0.27-1.32)	0.91 (0.49-1.81)	2.54 (2.30-3.47)*#
**miR-155**	2.38 (1.45-3.15)	1.90 (1.22-2.54)	0.79 (0.39-1.82)*°
**miR-424**	2.58 (2.49-3.13)	2.22 (1.74-3.26)	1.05 (0.80-2.31)§#

Data are shown as geometric mean (25^th^-75^th^ percentile). *p<0.01; §p<0.05 microalbuminuric diabetic patients vs non-diabetic controls. ° p<0.01; #p<0.05 micro- vs normoalbuminuric diabetic patients.

Previous studies in mesangial cells have shown that miR-145 is a target of TGF-β1 (21), a prosclerotic cytokine playing a key role in diabetic nephropathy; therefore, this miRNA was chosen for subsequent both *in vivo* and *in vitro* studies.

### Urinary exosomes from diabetic mice are enriched for miR-145

**Figure 1:**
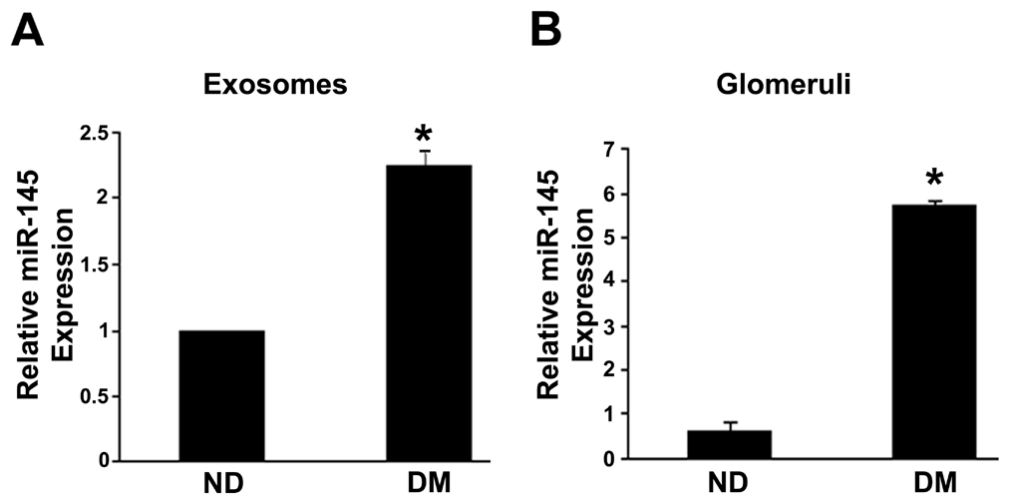
Expression of miR-145 in experimental diabetes. MiR-145 levels were measured by qRT-PCR in both urinary exosomes (**A**) and isolated glomeruli (**B**) from diabetic (DM) and control (ND) mice as described in the Methods. Results, corrected for the expression of housekeeping U6 snRNA, are shown in the graphs (*p<0.001 DM vs ND).

After 10 weeks of STZ-induced diabetes, both blood glucose and glycated hemoglobin levels were significantly higher in diabetic than in non-diabetic mice. Furthermore, compared with sham-injected control animals, diabetic mice showed a significant rise in albumin excretion rate (Table 3). Urinary exosomes were isolated from pooled urine from both diabetic and non-diabetic mice and miR-145 levels measured by qRT-PCR. Results showed that miR-145 levels were twice higher in diabetic than in non-diabetic animals (Figure 1A), indicating that urinary exosomes have a greater miR-145 content not only in human, but also in experimental DN. 

**Table 3:** General assessment parameters in diabetic and non-diabetic mice.

	**Non-Diabetic**	**Diabetic**
**N**	30	30
**Body weight (g)**	28.9 ± 0.41	24.9 ± 0.40*
**Blood Glucose (mg/dl)**	107.6 ± 4.21	347.5 ± 16.7*
**Glycated Hb (%)**	4.5 ± 0.12	11.6 ± 0.32*
**Systolic BP (mmHg)**	118 ± 2.98	120 ± 3.60
**AER (μg/18h)**	76.5 (60.4-89.2)	322.7 (218.9-348.1)*

Data are shown as mean ± SEM or geometric mean (25^th^-75^th^ percentile). BP: blood pressure; AER: albumin excretion rate; *p<0.001 diabetic vs. non-diabetic mice.

### miR-145 is overexpressed in the glomeruli from diabetic mice

To investigate if enhanced urinary exosomal miR-145 levels mirror a parallel change within the glomeruli, we studied miR-145 expression in glomeruli isolated from both diabetic and control mice. As shown in Figure 1B, miR-145 was weakly expressed in the glomeruli from non-diabetic mice, but highly expressed in the glomeruli from diabetic mice. Quantitatively, diabetic animals showed a significant nine-fold increase in glomerular miR-145 levels.

### Exposure to high glucose induces miR-145 expression in cultured mesangial cells

**Figure 2:**
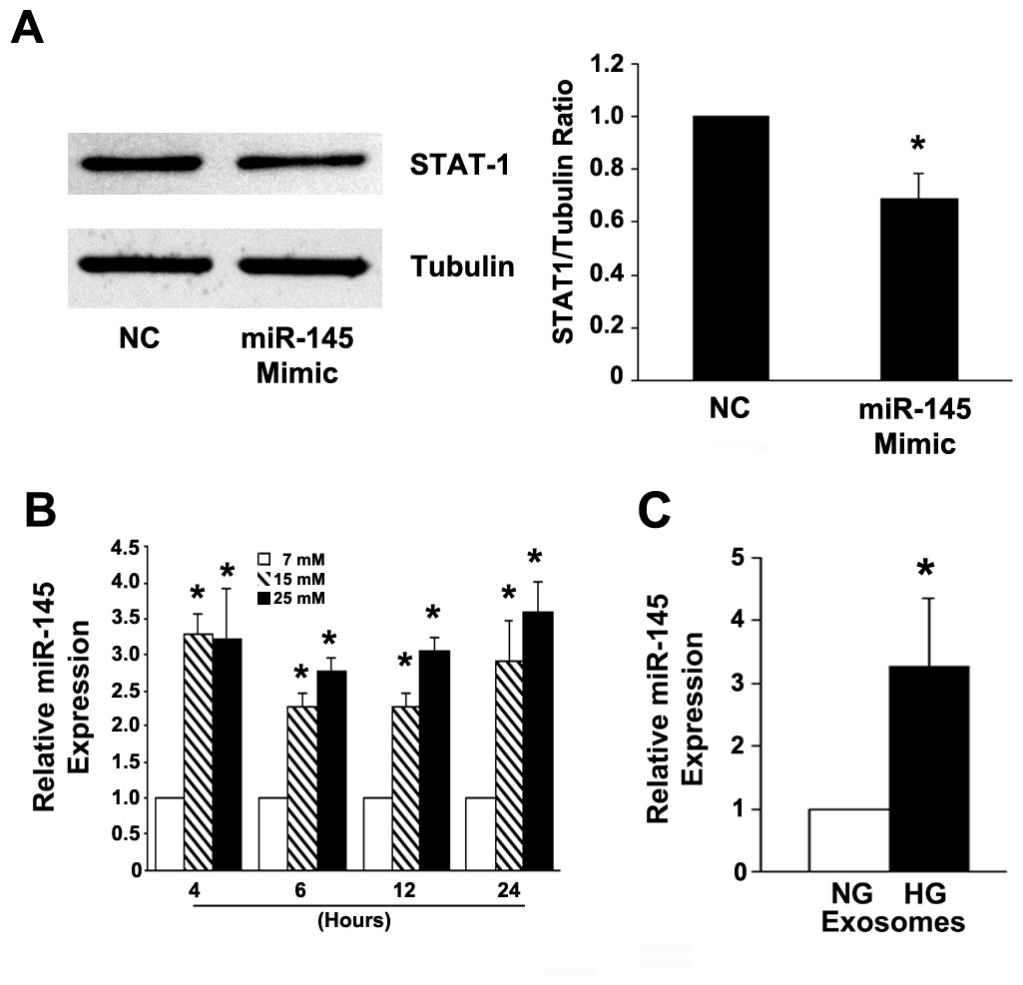
Expression of miR-145 in human mesangial cells. (**A**) Expression of STAT-1, a known miR-145 target, was assessed by immunoblotting in total protein extracts from mesangial cells transfected with either miR-145 mimic or scramble oligonucleotides. Tubulin was used as loading control. Results are depicted in the graph (*p<0.05) and a representative immunoblot is shown. (**B**) Human mesangial cells were exposed to increasing (6.8, 15, and 25 mM) glucose concentrations for 4,6,12 and 24 hours. Total RNA was extracted and levels of miR-145 measured by qRT-PCR (*p<0.01) 15 and 25 mM glucose vs. 6.8 mM glucose at all time points). (**C**) Human mesangial cells were exposed to either normal (6.8 mM-NG) or high (25 mM-HG) glucose concentrations for 24 hours. Exosomes were isolated from the supernatants by ultracentrifugation as described in the Methods. Total RNA was extracted and levels of miR-145 measured by qRT-PCR (*p<0.001 HG vs. NG).

Having established that diabetes induces glomerular miR-145 expression, we investigated the underlying cellular mechanism *in vitro* in mesangial cells. Hyperglycaemia is a key determinant in the pathogenesis of DN; therefore, we assessed if exposure to high glucose affected miR-145 expression in mesangial cells. MiR-145 was expressed by mesangial cells cultured in a normal glucose milieu. Furthermore, miR-145 was functionally active in this cell type as miR-145 transfection suppressed STAT-1 protein expression modestly, but significantly (Figure 2A). Exposure to both 15 and 25 mM glucose concentrations for 4 hours induced a significant three-fold rise in miR-145 levels and this effect was sustained up to 24 hours, suggesting that hyperglycemia may be implicated in glomerular miR-145 overexpression in diabetic mice (Figure 2B). 

### MiR-145 is enhanced in exosomes released by mesangial cell exposed to high glucose

To clarify if high glucose exposure also affects exosomal miR-145 content, exosomes were isolated from the supernatants of mesangial cells exposed for 24 hours to either high or normal glucose concentrations. Exposure to high glucose reduced the quantity of exosomes released by mesangial cells [NG: 1.28 10^7^/100000 cells (1.06-1.49) geometric mean (25^th^-75^th^ percentile); HG: 0.59 x 10^7^(0.58-0.60), p<0.05 NG vs HG], but significant enhanced exosomal miR-145 content (Figure 2C). Moreover, the high glucose-induced rise in exosomal miR-145 levels was comparable in magnitude to that observed in parent mesangial cells.

## Discussion

This study provides the first evidence that urinary exosomes contain a vast array of miRNAs. Urinary exosomes were obtained by a two-step differential ultracentrifugation process that is regarded as the gold standard method for exosomal isolation from biological fluids, including urine [14]. This method allows efficient isolation of exosomes with a high degree of purity, though it is laborious and time-consuming. Other larger types of vesicles may co-isolate with exosomes using most isolation methods; however, larger vesicles are unlikely to contribute significantly to our findings as isolated microvesicles were positive for specific exosomal markers and displayed both the size and shape characteristic of exosomes at the electron microscopy.

MiRNA expression profiling revealed a differential exosomal miRNA expression in two pairs of tightly matched micro/normoalbuminuric DM1 patients. Although these results are preliminary, they provide a proof of concept of the feasibility of this novel approach for biomarker discovery. Further studies are warranted to establish whether a distinctive urinary exosomal miRNA signature exists in DN and may be exploited as diagnostic/prognostic tool. It is noteworthy in this regard that our patients had low degree of microalbuminuria and normal renal function, suggesting that abnormalities in urinary exosomal miRNA expression may occur in an early stage of the disease. 

In diabetic patients with microalbuminuria there was also a trend toward a reduction in urinary exosome quantity. Although the difference did not reach statistical significance, this finding may be of potential relevance and worth of further investigations in larger series of patients as exosome count may represent an additional aspect of the potential use of exosome as DN biomarker. 

A recent paper by Argyropoulos C et al has reported changes of urinary miRNA spectrum in DM1 patients with different stages of albuminuria and nephropathy [22]. Differentially expressed miRNAs identified in this report do not overlap with those found in our study. This is, however, not surprising because Argyropoulos C et al. compared urine miRNAs from DM1 patients with persistent and intermittent microalbuminuria and profiled total urinary miRNAs rather than exosomal miRNAs. Although total urine miRNA profiling is easier to perform and less time-consuming with consequent advantages for translation in clinical practise, the analysis of exosomal urinary miRNAs is likely to be more accurate as total urine miRNAs can also originate from apoptotic/necrotic cells facing the urinary tract. In addition, as plasma miRNAs have a small molecular weight and can pass the glomerular filtration barrier, a substantial portion of total urine miRNAs is likely of plasma rather than of renal origin and this diminishes the relevance of total urinary miRNA analysis for biomarker discovery in renal pathophysiology.

Our profiling results did not reveal significant difference in miR-192 expression between micro and normoalbuminuric DM1 patients, though miR-192 has been implicated in the pathogenesis of DN and is induced by TGF-β1 in mesangial cells [23]. Opposing modulation of miR-192 in mesangial cells [23] and tubular epithelial cells in DN [24], resulting in an overall null effect on urinary miRNA levels may be a potential explanation. Alternatively, we cannot exclude the possibility that cells overexpressing miR-192 release exosomes with normal miR-192 content as exosomes carry exclusively miRNAs that need to be deliver to distant target cells.

Validation of profiling results in all recruited patients confirmed that urinary exosomal miR-145 levels were significantly enhanced in microalbuminuric as compared to normoalbuminuric DM1 patients and control non-diabetic subjects. This finding is of potential pathophysiological relevance as miR-145 is a glomerular marker of mesangial cells [20] and is induced by TGF-β1 in this cell type [21]. In keeping with this hypothesis, our in vivo study showed that miR-145 levels were increased in both the urinary exosomes and the glomeruli isolated from streptozotocin-induced diabetic mice. This is the first report of glomerular miR-145 overexpression in an experimental model of DN and together with previous studies, reporting altered miRNA expression in DN [25,26], support the hypothesis of a miRNA involvement in the development of the complication. 

*In vitro* in cultured mesangial cells miR-145 was expressed and functionally active as its overexpression resulted in suppression of STAT-1, a known miR-145 target [27]. Exposure to high glucose induced a rapid and sustained increase in miR-145 expression in human mesangial cells, suggesting hyperglycaemia as a possible insult inducing glomerular miR-145 overexpression. TGF-β1 is a likely mediator because high glucose is a well established TGF-β1 inducer in mesangial cells [28] and TGF-β1 rises miR-145 expression in both mesangial [21] and vascular smooth muscle cells through a SMAD-dependent pathway [29]. Recent studies in vascular muscle cells have proven that miR-145 is a key determinant in the switching from a proliferative to a differentiated/contractile phenotype [19,30], raising the hypothesis of a miR-145 role in high glucose-induced mesangial cell both hypertrophy and cytoskeleton remodelling.

In diabetic mice, glomerular miR-145 overexpression was paralleled by an increase in urinary exosomal miR-145 content. In addition, mesangial cell exposure to high glucose induced the release of exosomes enriched in miR-145, mirroring the overexpression observed in parent cells. This raises the attractive possibility of a mesangial cell origin of urinary exosomes carrying enhanced miR-145 content. Although exosome size is over the cut-off value for glomerular filtration, a recent study has proven that exosomes can cross physiological barriers, such as the brain blood barrier [31]. The underlying mechanism is unclear; however, a process of transcytosis, possibly through the multivesicular bodies (MVB) compartment, has been proposed [32,33]. 

In various cell types exosome release is enhanced by cellular stresses [34]; however, in our study the quantity of exosomes released by mesangial cells exposed to high glucose, a known inducer of oxidative stress, was significantly diminished and could not account for the rise in miR-145 levels. The underlying mechanism is unknown; however, it is tempting to speculate that high glucose may interfere in the complex process leading to exosome formation/release from the MVB. 

Although our *in vivo* and *in vitro* work points to hyperglycaemia as a likely miR-145 inducer; HbA1c levels were similar in DM1 with and without microalbuminuria and do not justify the greater miR-145 exosomal content seen in patients with incipient DN. However, we cannot exclude the possibility that serum glucose levels differ in the two groups at the time of urine sampling. In addition, in the microalbuminuric group greater blood glucose fluctuation in the period prior to sampling and/or enhanced formation of advance glycation end-products/TGF-β1 may provide explanation for a glucose-dependent effect despite similar HbA1c levels. Finally, patients in the microalbuminuric group are likely to have experienced in the past poor metabolic control and a metabolic memory-related effect cannot be rule out. Consistent with the hypothesis of a glucose-related effect, miR-145 values were also enhanced in the urinary exosomes from normoalbuminuric DM1 patients as compared to controls, though this did not reached statistical significance.

Urinary exosomal miR-130a was also up-regulated in DM1 patients and further increased in subjects with microalbuminuria. On the contrary, miR-155 and miR-424 were downregulated and this effect was observed specifically in DM1 with incipient DN. These miRNAs are known to be expressed, though not exclusively, by podocytes and podocyte-derived exosomes can be easily found into urine as this cell type faces the urinary space. Of interest, recent studies have shown that miR-155 and miR-424 negatively modulate the signalling of cytokines, such as angiotensin II [35,36], TGF-β1 [37,38], and VEGF [39], that play a key role in the pathogenesis of DN. Whether changes in the urinary exosomal content of miR-130a, miR-155, miR-424 mirror parallel changes in parent cells remains, however, to be established.

In conclusion, in this study we have identified miR-145 as a new potential player in diabetic glomerulopathy and demonstrated the feasibility of the study of urinary exosomal miRNA for candidate biomarker discovery in diabetic and other renal diseases. Further studies on larger series of patients are warranted to confirm our findings and assess their clinical relevance.

## Supporting Information

Figure S1 **Identification and characterisation of exosomes.** (**A**) Urinary exosomes were isolated from overnight urine collections by differential ultracentrifugation as describe in the Methods. Urinary vesicles, showing the characteristic exosomal cup-shape and size, are shown in the representative electron micrograph; scale bar = 200nm. (**B**) Expression of both exosomal (Hsp70, alix) and endoplasmatic reticulum (calnexin) specific markers was assessed by immunoblotting in total protein extracts from isolated urinary exosomes and positive controls (MC: mesangial cells). GAPDH was used as loading control for calnexin. Representative immunoblots are shown. Total RNA, extracted from urinary exosomes, was analyzed on an Agilent 2100 Bioanalyzer using either a RNA 6000 Pico Kit (**C**) or a small RNA kit (**D**). The representative electropherogram shows that urinary exosomes contain small RNA species (<150 nt), including microRNAs (10-40 nt).
(TIF)
