# Supplementary figures and images for: Urinary Exosomal MicroRNAs in Incipient Diabetic Nephropathy
Source: PLoS One. 2013 Nov 4;8(11):e73798. doi: 10.1371/journal.pone.0073798 (PMC3817183; doi:10.1371/journal.pone.0073798)

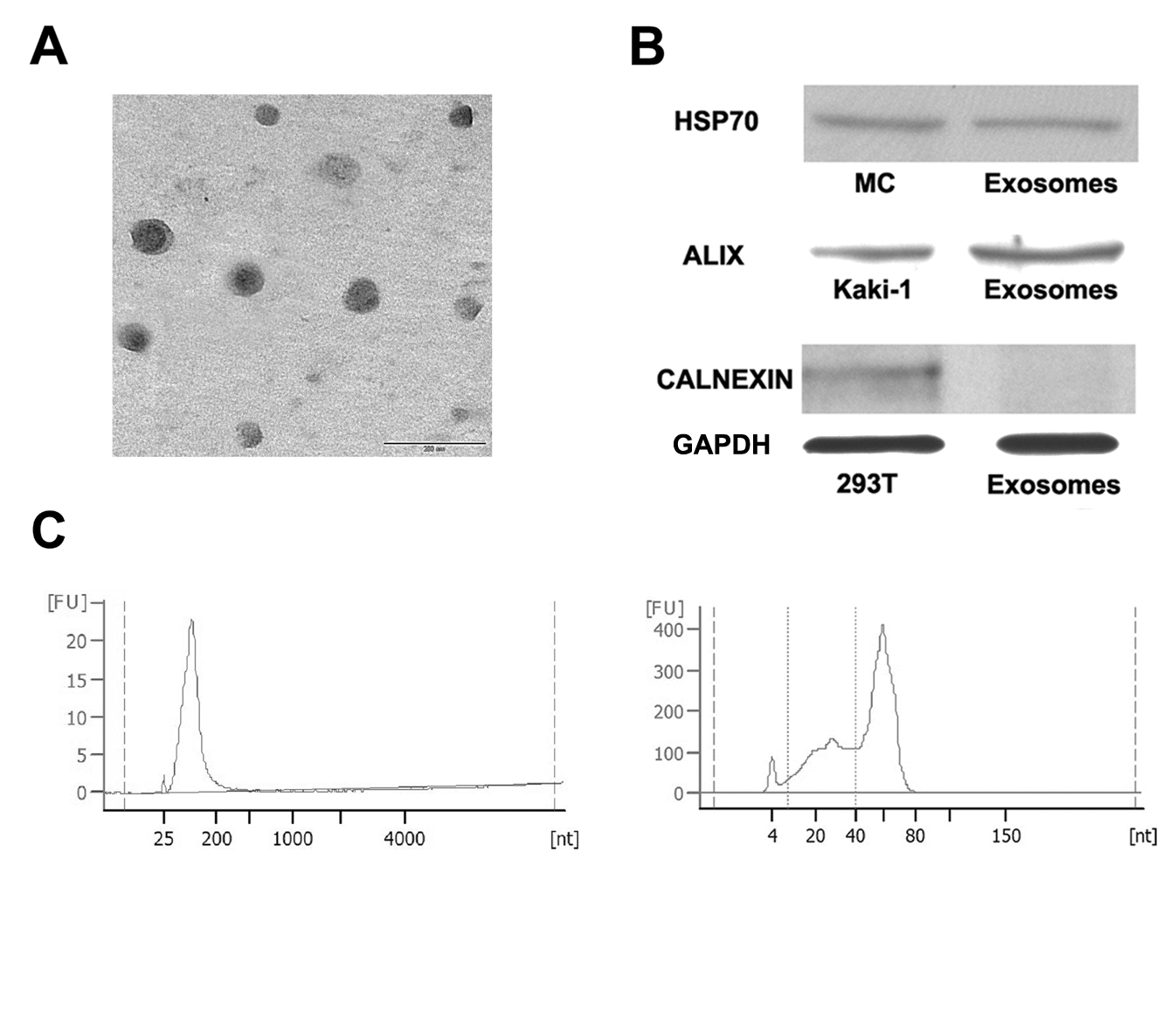

Supplement: Figure S1 — Identification and characterisation of exosomes. (A) Urinary exosomes were isolated from overnight urine collections by differential ultracentrifugation as describe in the Methods. Urinary vesicles, showing the characteristic exosomal cup-shape and size, are shown in the representative electron micrograph; scale bar = 200nm. (B) Expression of both exosomal (Hsp70, alix) and endoplasmatic reticulum (calnexin) specific markers was assessed by immunoblotting in total protein extracts from isolated urinary exosomes and positive controls (MC: mesangial cells). GAPDH was used as loading control for calnexin. Representative immunoblots are shown. Total RNA, extracted from urinary exosomes, was analyzed on an Agilent 2100 Bioanalyzer using either a RNA 6000 Pico Kit (C) or a small RNA kit (D). The representative electropherogram shows that urinary exosomes contain small RNA species (<150 nt), including microRNAs (10-40 nt). (TIF) [file pone.0073798.s001.tif]
